# The Enhancement of Abiotic Stress Tolerance in *Arabidopsis* via Heterologous Overexpression of *TcDHN1*, a Dehydrin Identified in the Recalcitrant Seeds of *Taxillus chinensis*

**DOI:** 10.3390/plants15060884

**Published:** 2026-03-12

**Authors:** Ya Qin, Yuqiong Li, Cuihong Yang, Wenjing Liang, Lingjian Gui, Lisha Song, Jie Shen, Ru Chen, Limei Pan, Shugen Wei, Lingyun Wan

**Affiliations:** 1 Guangxi Key Laboratory for High-Quality Formation and Utilization of Dao-di Herbs, Guangxi Botanical, Garden of Medicinal Plants, Nanning 530023, China; qygxyyzwy@163.com (Y.Q.);; 2 National Center for Traditional Chinese Medicine Inheritance and Innovation, Guangxi Botanical, Garden of Medicinal Plants, Nanning 530023, China; 3 National Engineering Research Center for the Development of Southwestern Endangered Medicinal Materials, Guangxi Botanical, Garden of Medicinal Plants, Nanning 530023, China

**Keywords:** *Taxillus chinensis* (DC.) Danser, recalcitrant seeds, TcDHN1, heterologous overexpression, antioxidant enzymes, abiotic stress tolerance

## Abstract

*Taxillus chinensis* (DC.) Danser is an important hemiparasitic medicinal plant whose propagation is severely limited by the desiccation sensitivity of its recalcitrant seeds. Dehydrins (DHNs), which protect plants against dehydration-induced stresses such as salinity, drought, and low temperatures, may play a critical role in protecting recalcitrant seeds. However, the role of DHNs in the seeds of *T. chinensis* remains unclear. In this study, a differentially expressed gene was identified from the seed transcriptome of *T. chinensis* and designated *TcDHN1.* Sequence alignment and phylogenetic analyses revealed that *TcDHN1* encodes a dehydrin protein. Heterologous overexpression of *TcDHN1* in *Arabidopsis* did not affect growth under normal conditions. Under salt, drought, and cold stresses, transgenic lines exhibited higher seed germination rates, longer primary roots, and improved seedling growth compared with wild-type (WT) plants. The transgenic lines showed significantly increased activities of antioxidant enzymes, including superoxide dismutase, catalase, and peroxidase. In addition, ectopic overexpression of *TcDHN1* in *Arabidopsis* conferred enhanced tolerance to abiotic stresses compared to WT plants, accompanied by increased expression of the stress-responsive genes Responsive to Desiccation 29A (*AtRD29A*) and Heat Shock Protein 70-1 (*AtHSP70-1*). The above results indicate that *TcDHN1* confers enhanced tolerance to abiotic stresses. This study provides a functional characterization of an abiotic stress-responsive gene from recalcitrant seeds and identifies a potential genetic resource for molecular breeding. This could potentially improve abiotic stress resistance in *T. chinensis* and related medicinal plants.

## 1. Introduction

Abiotic stresses, including extreme temperatures, drought, and salinity, can severely limit plant growth, development, and productivity [1]. In response, plants activate a complex array of physiological and molecular defenses mechanisms, including the synthesis of protein kinases, antioxidant enzymes, and late embryogenesis abundant (LEA) proteins [2,3]. Among these components, LEA proteins, such as the dehydrins (DHNs) subfamily, play a critical role in plant resistance to various abiotic stresses [4,5]. DHN is a type of hydrophilic protein widely found in plants, and they typically contain 82 to 575 amino acids [6]. The molecular weights of DHNs varies considerably across different forms, ranging from approximately 9 to 200 kDa [7,8]. A defining structural feature of dehydrins is the presence of three highly conserved regions, including the K-segment (consensus sequence XKXGXX(D/E)KIK(D/E)KXPG), the Y-segment (commonly D(D/E/Q)(Y/H/F)GNP), and the S-segment (typically comprising 4–6 tandem serines, represented as SSSSSED) [9,10]. Based on their conserved segments, DHNs are usually classified into five subfamilies, which are KnS, YnSKn, Kn, YnKn, and SKn [11,12]. Heterologous overexpression of DHN genes from various species has been shown to confer improved tolerance to cold temperatures, drought, and salinity, demonstrating their conserved protective potential. For example, heterologous overexpression of the *Saussurea involucrata* SiDHN in tomato, improves its tolerance to drought conditions [13]. Similarly, heterologous expression of *Jatropha curcas JcDHN2* in rice enhances its tolerance to water stress [14]. Likewise, overexpression of the pepper *CaDHN4* in *Arabidopsis thaliana* alleviates damage caused by low temperature and salt stress [15].

*Taxillus chinensis* (DC.) Danser is a hemiparasitic species in the family Sapindaceae and is used in many traditional Chinese medicine preparations. It is used for treating conditions described as “dispelling wind and dampness”, nourishing the liver and kidneys, strengthening muscles and bones, and preventing miscarriage. It has been listed in previous editions of the Chinese Pharmacopoeia [16], and is considered a representative medicinal resource of Guangxi Province, China. However, its cultivation and conservation are severely constrained by biological factors. The species relies exclusively on seeds for propagation, although these are recalcitrant and highly sensitive to dehydration, resulting in rapid loss of viability. Recalcitrant seeds are characterized by having high desiccation sensitivity and they rapidly lose viability under conditions that promote cellular dehydration [17]. Abiotic stresses such as drought, high salinity, and low temperature primarily inflict damage by disrupting cellular water balance and causing severe dehydration, which mirrors the natural vulnerability of these seeds [18]. This trait severely limits germplasm preservation, seed storage, and subsequent seedling development, thereby hindering their large-scale cultivation and production [19]. To date, most studies on *T. chinensis* have focused on its chemical constituents and pharmacological activities [20,21]. *T. chinensis* predominantly grows in subtropical regions with seasonal temperature fluctuations. Although low temperatures are commonly used for seed storage [19], they are detrimental to recalcitrant seeds because freezing induces cellular dehydration. Consequently, the molecular mechanisms underlying seed desiccation sensitivity and intrinsic stress tolerance remain largely unclear. Elucidating these mechanisms, especially the role of protective proteins such as DHNs, is essential for developing effective strategies to overcome this propagation limitation.

Compared with conventional hybrid breeding, transgenic technology offers shorter breeding cycles, greater genetic stability, and precise improvement of target traits, thereby providing a predictive molecular approach for developing stress-resistant medicinal plants such as *T. chinensis* [22]. However, the absence of an established genetic transformation system for *T. chinensis* makes it difficult to validate gene function in this species directly. The model plant *Arabidopsis*, which is characterized by a short life cycle, a fully sequenced genome, and a relatively high conservation of gene function across plant species, provides an efficient heterologous system for functional validation. Based on transcriptomic analysis of *T. chinensis* seeds, we identified a candidate dehydrin gene, *TcDHN1*, that was differentially expressed under stress conditions [19,23]. We hypothesized that *TcDHN1* contributes to the ability of *T. chinensis* to tolerate abiotic stresses. This study aimed to isolate the *TcDHN1* gene and functionally characterize its role in abiotic stress responses. An overexpression vector, pCAMBIA1301-*TcDHN1*, was constructed, and transgenic *A. thaliana* lines were generated. The common link between drought, salinity, and cold stress is cellular dehydration, which is the primary threat to recalcitrant seed viability. Therefore, we evaluated the key phenotypic traits (germination, root growth, and seedling survival) as well as physiological indicators such as antioxidant enzyme activities of the transgenic lines under these stress conditions. This study provides the first functional evidence linking *TcDHN1* to abiotic stress tolerance. Our findings will elucidate potential adaptive mechanisms in recalcitrant-seeded *T. chinensis* seeds. In addition, we identified *TcDHN1* as a promising genetic resource for biotechnological strategies aimed at enhancing abiotic stress tolerance and overcoming the propagation barriers encountered by this species.

## 2. Results

### 2.1. Sequences and Phylogenetic Analyses

**Figure 1:**
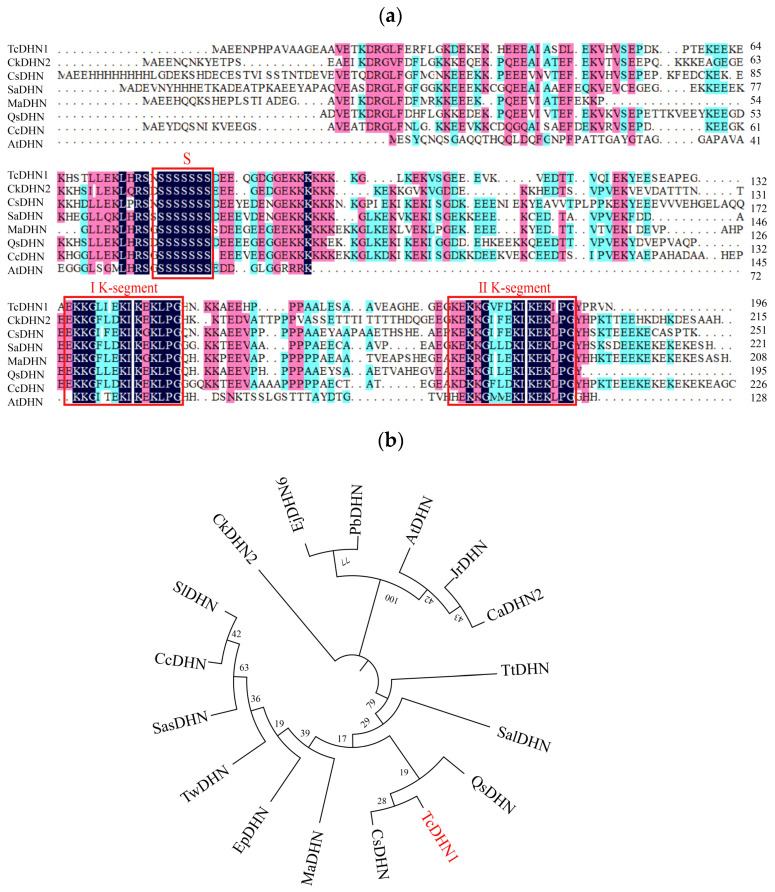
Identification of the *TcDHN1* gene. (**a**) Amino acid sequence alignment of TcDHN1 protein. Red squares indicate conserved amino acid sequences of S- and K-segment motifs; (**b**) Phylogenetic relationships between the TcDHN1 protein and dehydrins (DHNs) from other plant species. The molecular phylogeny was constructed from complete protein sequence alignment using the neighbor-joining method with MUSCLE. TcDHN1 is highlighted in red in the phylogenetic tree. The following information (species, gene names, and GenBank accession numbers) on the DHN proteins were obtained: TcDHN1 (*Taxillus chinensis*, PX833254), CkDHN2 (*Caragana korshinskii*, AHK05942.1), CsDHN (*Camellia sinensis*, ACT10283.1), SasDHN (*Striga asiatica*, GER39990.1), MaDHN (*Melia azedarach*, KAJ4702232.1), QsDHN (*Quillaja saponaria*, KAJ7966939.1), CcDHN (*Coffea canephora*, ABC68275.1), AtDHN (*Arabidopsis thaliana*, AAB00375.1), SalDHN (*Santalum album*, XDC68618.1), TtDHN (*Thalictrum thalictroides*, KAF5181770.1), CaDHN (*Capsicum annuum*, KAF3667866.1), EjDHN (*Euphorbia peplus*, WCJ44327.1), TwDHN (*Tripterygium* wilfordii, XP 038715479.1), SlDHN (*Solanum lycopersicum*, NP001316365.1), EjDHN6 (*Eriobotrya japonica*, AGV21055.1), PbDHN (*Pyrus bretschneideri*, XP009346053.1), and JrDHN (*Juglans regia*, AGJ94409.1).

The full-length sequence (CDS) of *TcDHN1* was successfully cloned from *T. chinensis* (Figure S1). The CDS comprised 590 bp and encoded a protein of 196 amino acids (Figure S2), with a predicted molecular weight of 21.67 kDa and an isoelectric point of 5.38. The average hydrophilicity index of TcDHN1 was −1.339, indicating that the protein is highly hydrophilic (Figure S3). Multiple sequence alignments shown in Figure 1a indicated that TcDHN1 shared a high similarity with DHN proteins from other plants species (Figure 1a). Comparative sequence analysis with revealed that TcDHN1 contains two K-segments and one S-segment, classifying it as an SK2-type DHN within the SKn subfamily. Phylogenetic analysis (Figure 1b) showed that *TcDHN1* clustered closely with *CsDHN* from *Camellia sinensis*, suggesting a close evolutionary relationship.

### 2.2. Vector Construction and Genetic Transformation of Arabidopsis Lines

The overexpression vector was constructed as shown in Figure S4. Briefly, the CDS of *TcDHN1* was amplified from *T. chinensis* cDNA using primers containing AbsI and KpnI restriction sites (Table S1), generating a fragment of approximately 590 bp. The purified PCR product was ligated into the linearized pCAMBIA1301 vector and transformed into *Escherichia coli* DH5α. The recombinant plasmid was verified by restriction digestion, releasing fragments corresponding to the linearized vector (~10 kb) and it was then inserted into the *TcDHN1* CDS (~590 bp), which is consistent with the expected sizes (Figure S2). The insert sequence was further confirmed by DNA sequencing. The confirmed recombinant plasmid, designated pCAMBIA1301-TcDHN1, was introduced into the *Agrobacterium tumefaciens* strain, GV3101, by using the freeze–thaw method.

**Figure 2:**
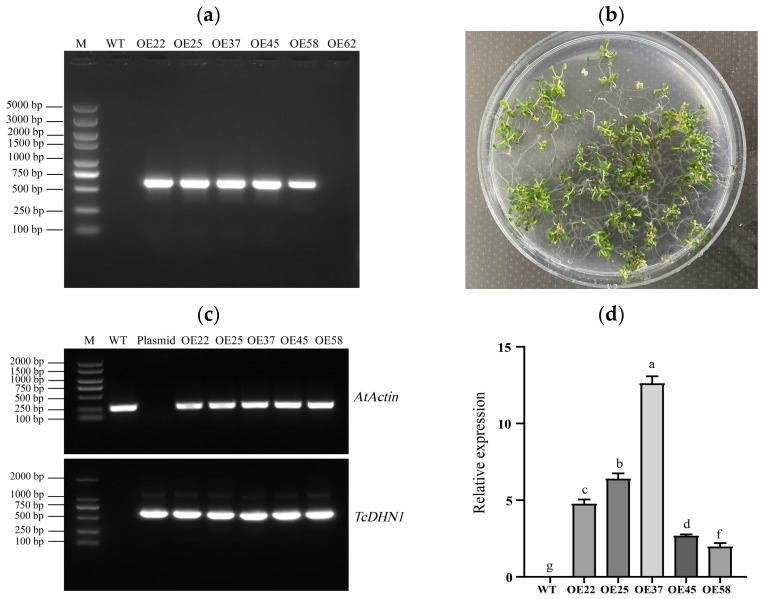
Identification of T1 and T2 generation of *Arabidopsis* plants transformed with the *TcDHN1* gene. (**a**) Detection of T1 generation *Arabidopsis* DNA after transformation with *TcDHN1*; M: DNA 2000 Marker; WT: wild-type *Arabidopsis* sample; OEs: the samples were obtained from putative positive transgenic lines; (**b**) Segregation ratio analysis of T1 generation TcDHN1-positive *Arabidopsis*; (**c**) Semi-quantitative analysis of T2 generation *Arabidopsis*; (**d**) Relative expression levels of the *TcDHN1* gene in T2 generation transgenic *Arabidopsis*. The values are obtained from triplicate determinations and are represented as means ± SE. The different letters above the bars specify significant differences (*p* < 0.05) as assessed with Tukey’s test.

Wild-type (WT) seeds were transformed via *Agrobacterium* and sown on kanamycin-containing MS medium. Six resistant T1 seedlings were obtained. PCR amplification of the genomic DNA yielded a specific band (~590 bp) in five of the six resistant seedlings, verifying them as positive transformants (Figure 2a). The confirmed T1 plants were grown to maturity and self-pollinated. Segregation analysis of T2 progeny on kanamycin-containing MS medium showed an approximately 3:1 ratio of resistant to susceptible seedlings, indicating that the transgene was inserted at a single T-DNA locus (Figure 2b).

Semi-quantitative RT-PCR analysis of T2 plants detected amplification of both the *TcDHN1* target band and the *AtActin* internal control, confirming transcription of the transgene (Figure 2c). Quantitative real-time PCR (qRT-PCR) revealed significantly higher TcDHN1 transcript levels in five independent T2 homozygous lines (OE22, OE25, OE37, OE45, and OE58) compared with WT plants (Figure 2d). Among these, OE22, OE25, and OE37 exhibited the highest expression levels and consistent phenotypic performance in preliminary screenings, and these were therefore selected for subsequent abiotic stress tolerance assays.

### 2.3. Overexpression of TcDHN1 Enhances Salt Tolerance in Arabidopsis

**Figure 3:**
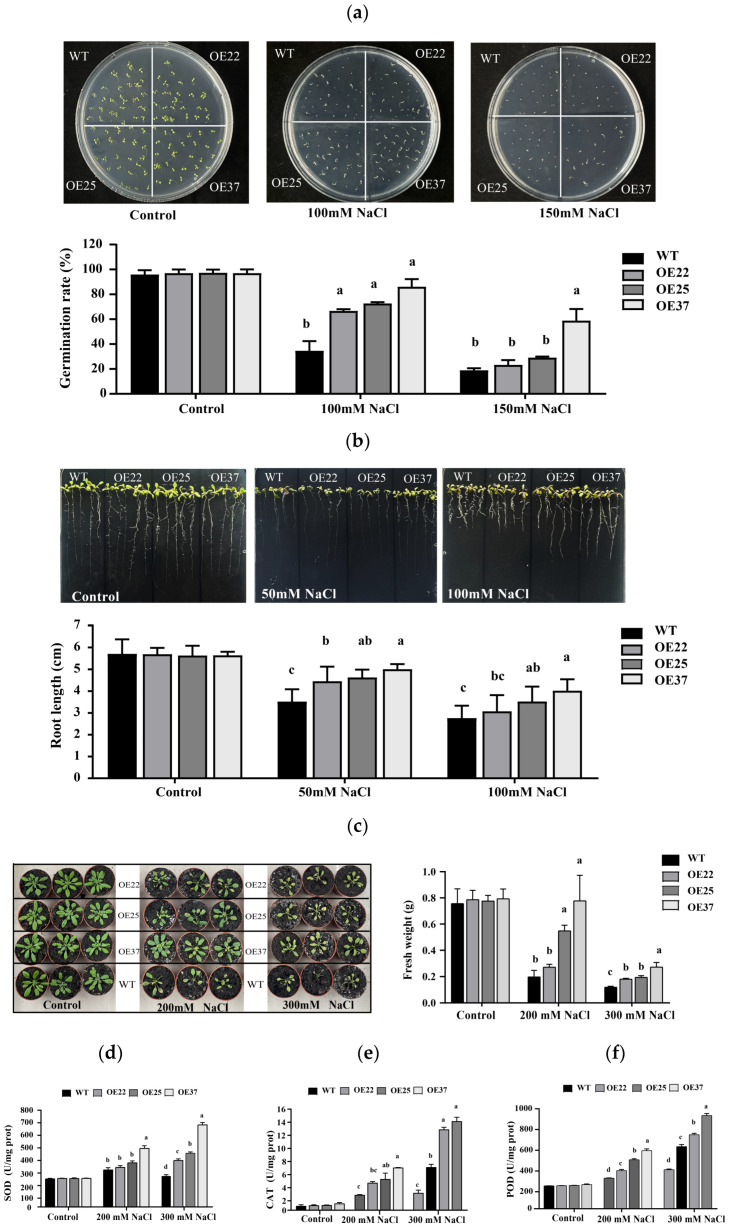
The performance of WT and *TcDHN1*-OEs transgenic plants when subjected to salt stress. (**a**) The germination phenotype and rate analyses of WT and *TcDHN1*-OEs transgenic plants in the presence of 100 mM NaCl and 150 mM NaCl stresses; (**b**) The root lengths of WT and *TcDHN1*-OEs transgenic plants subjected to 50 mM NaCl and 100 mM NaCl stresses; (**c**) The growth phenotypes and fresh weights of WT and *TcDHN1*-OEs transgenic plants subjected to 200 mM NaCl and 300 mM NaCl stresses; (**d**–**f**) The enzymatic activities of SOD, CAT and POD in the WT and *TcDHN1*-OEs transgenic plants under natural and salt stresses. The values are obtained from triplicate determinations and are represented as the means ± SEMs. The different letters above the bars specify significant differences (*p* < 0.05) as assessed with Tukey’s test.

To evaluate salt stress conferred by *TcDHN1* overexpression, three transgenic lines (OE22, OE25, and OE37) and WT plants were examined at early developmental stages. Under normal MS medium conditions, all lines exhibited germination rates of approximately 99%, and no significant difference in seedling root length (approximately 5.6 cm) were observed, indicating that *TcDHN1* overexpression did not affect basal growth (Figure 3a,b). Salt stress significantly inhibited seed germination and root elongation in a concentration-dependent manner. Under 100 mM and 150 mM NaCl stress, the germination rates of the transgenic lines were consistently higher than those of WT plants. At 150 mM NaCl, the germination rate of the OE37 reached 58.6%, significantly higher than the 18.6% observed with the WT (Figure 3a). Root length analysis (Figure 3b) showed that increasing NaCl concentrations markedly inhibited root elongation in all lines and caused abnormal leaf morphologies. Compared with WT plants, *TcDHN1*-overexpressing lines exhibited less severe inhibition of root elongation and maintained more normal leaf morphology. In addition, under 150 mM NaCl stress, the root lengths of the OE25 and OE37 lines were significantly greater than of the WT.

To further characterize the performance of *TcDHN1*-overexpressing lines under salt stress, soil-grown seedlings were subjected to salt stress by irrigation with NaCl solutions. Under 200 mM and 300 mM NaCl stresses, all plants exhibited typical salt injury symptoms, including growth retardation and leaf chlorosis. However, the transgenic lines were less severely affected. As shown in Figure 3c, under 300 mM NaCl stress, the WT plants exhibited severe wilting, leaf abscission, and extensive necrosis, whereas most transgenic lines maintained relatively normal morphology, with OE37 displaying the strongest phenotype. Fresh weight measurements indicated that the transgenic lines had significantly higher biomass than the WT under both salt concentrations.

The improved phenotypic salt stress tolerance was associated with a stronger physiological enzyme activities (Figure 3d–f). Antioxidant enzyme activities (SOD, CAT, and POD) were higher in the transgenic lines than in the WT, indicating a more effectively activated antioxidant defense system. Taken together, these results from germination, root growth, seedling growth status, and physiological defense consistently demonstrate that overexpression of the *TcDHN1* gene significantly enhanced the overall salt tolerance capacity of *Arabidopsis*.

### 2.4. Overexpression of TcDHN1 Enhances Cold Tolerance in Transgenic Arabidopsis

**Figure 4:**
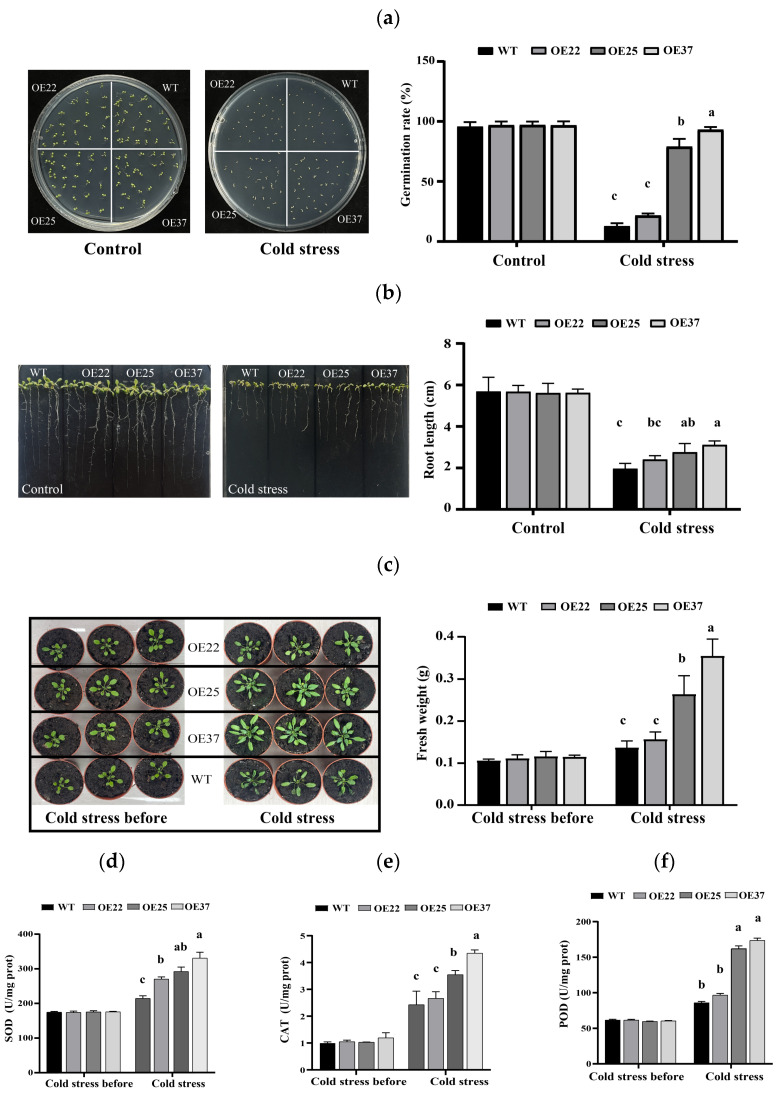
The performance of WT and OEs transgenic plants when subjected to low-temperature stress. (**a**) The germination phenotypes and rate analyses of WT and OEs transgenic plants subjected to low-temperature stresses. (**b**) The root lengths of WT and OEs transgenic plants subjected to low-temperature stresses. (**c**) The growth phenotypes and fresh weight of WT and OEs transgenic plants subjected to low-temperature stresses. The enzymatic activities of SOD, CAT, and POD in the WT and OEs transgenic plants under natural and low-temperature stress (**d**–**f**). The values are obtained from triplicate determinations and are represented as the means ± SEMs. The different letters above the bars specify significant differences (*p* < 0.05) as assessed with Tukey’s test.

To verify the functional capacities of *TcDHN1*-overexpressing lines under low-temperature stress, the identified lines (OE22, OE25, OE37) and WT plants were assessed for cold tolerance. Under normal growth conditions on MS medium, the seed germination and seedling growth capacities showed non-significant differences between the WT and transgenic lines (Figure 4a,b). However, at 4 °C, the seed germination was significantly inhibited in the WT, while the *TcDHN1*-overexpressing lines maintained substantially higher germination rates (Figure 4a). Specifically, the germination rates of OE25 and OE37 were 78.8% and 93.0%, respectively, significantly higher than that of the WT. Similarly, root growth under cold stress was less inhibited in the *TcDHN1*-overexpressing group compared to the WT (Figure 4b). When three-week-old soil-grown plants were exposed to 4 °C in a growth chamber for two weeks, growth of the WT plants was severely inhibited, exhibiting cold damage phenotypes such as stunted growth and leaf wilting. In contrast, the transgenic lines, particularly OE25 and OE37, sustained only mild damage, most of the leaves remained green, and the plants continued to grow, which correlated with their significantly higher fresh weights (Figure 4c).

SOD, CAT, and POD activities were similar across all the lines before cold treatment. However, upon exposure to low temperature, the *TcDHN1*-overexpressing lines exhibited significantly higher enzyme activities (Figure 4d–f), indicating an enhanced protective capacity against oxidative damage induced by freezing stress. This physiological response was consistent with the observed cold-tolerant phenotypes of the transgenic lines. Therefore, in general, the overexpression of *TcDHN1* enhanced cold tolerance in *Arabidopsis*, which is associated with the maintenance of better growth status, higher biomass accumulation, and a more robust antioxidant protection system.

### 2.5. Overexpression of TcDHN1 Enhances Drought Tolerance in Arabidopsis

**Figure 5:**
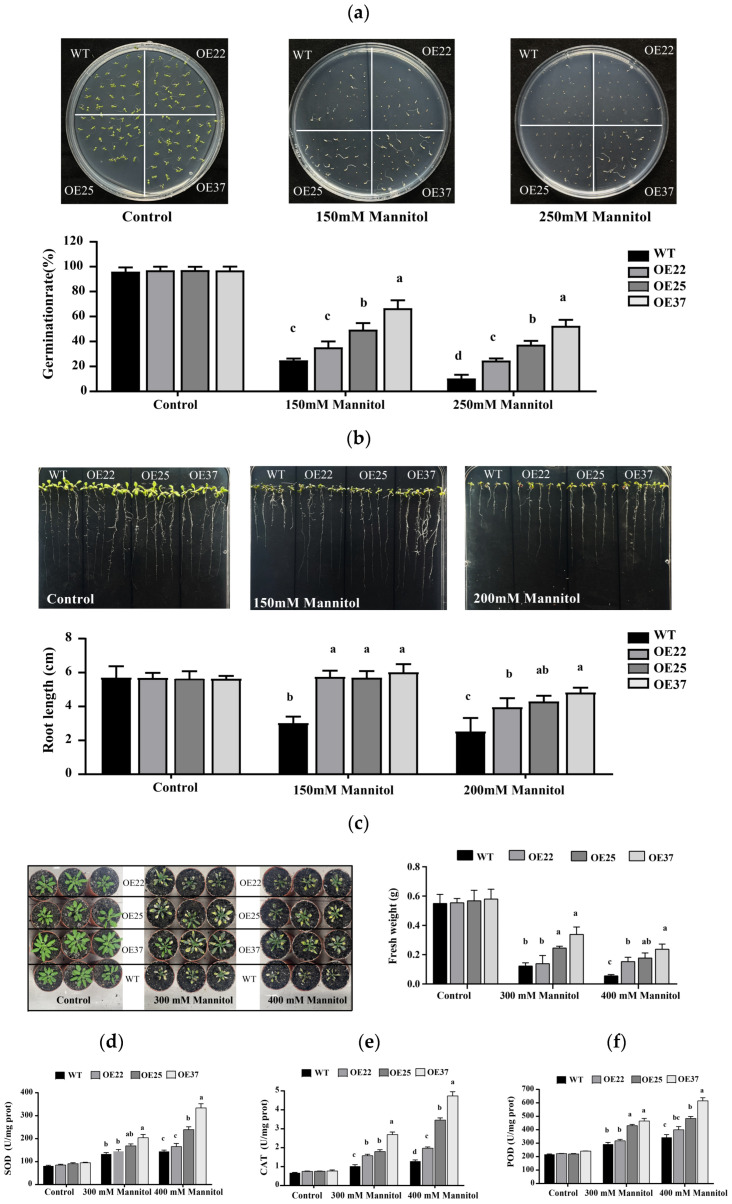
The performance of WT and *TcDHN1*-OEs transgenic plants when subjected to drought stress. (**a**) The germination phenotypes and rate analyses of WT and *TcDHN1*-OEs transgenic plants subjected to drought stress; (**b**) The root lengths of WT and *TcDHN1*-OEs transgenic plants subjected to drought stress; (**c**) The growth phenotypes and fresh weights of WT and *TcDHN1*-OEs transgenic plants subjected to drought stress; (**d**–**f**) The enzymatic activities of SOD, CAT and POD in the WT and *TcDHN1*-OEs transgenic plants under natural and drought stress. The values are obtained from triplicate determinations and are represented as the means ± SEMs. The different letters above the bars specify significant differences (*p* < 0.05) as assessed with Tukey’s test.

The response of the OE25, OE37, OE45 transgenic lines to drought stress as simulated by exposure to mannitol was evaluated. Under control conditions, the seed germination and seedling growth were comparable between transgenic lines and WT (Figure 5a,b). Following treatment with 150 mM and 250 mM mannitol, the seed germination was inhibited in all lines, but the transgenic lines, particularly OE25 and OE37, exhibited significantly higher final germination rates when compared to the WT (Figure 5a). Similarly, root lengths were less inhibited in the transgenic lines, specifically, at 150 mM and 200 mM mannitol, when the OE37 line exhibited increased lengths 3.0 cm and 2.3 cm respectively, when compared to the WT (Figure 5b).

Three-week-old soil-grown WT and transgenic lines were subjected to drought stress via irrigation with different concentrations of mannitol. Both WT and transgenic lines grew well with healthy green phenotypes under normal conditions (Figure 5c). After treatment with 300 and 400 mM mannitol, all the plants exhibited varying degrees of leaf yellowing. Under 300 mM mannitol irrigation, the three transgenic lines maintained partially green leaves and showed less growth inhibition, whereas the WT plants exhibited greater levels of growth retardation and leaf yellowing. The fresh weights of all transgenic lines were higher than that of the WT, with OE25 and OE37 showing significant increases of 0.1232 g and 0.2165 g, respectively, when compared to the WT. After 400 mM mannitol treatment, the WT plants displayed pronounced leaf yellowing, wilting, and even death, while the transgenic lines also suffered stress, but showing slower growth and leaf yellowing. In addition, their fresh weights remained higher than that of the WT plants.

Under healthy growth conditions, the activities of SOD, CAT, and POD were not markedly different among the various lines (Figure 5d–f). However, following drought stress treatment, the SOD, CAT, and POD activities in the transgenic lines were higher than those in the WT plants. Overall, the results demonstrated that overexpression of the *TcDHN1* gene significantly enhanced the tolerance of *Arabidopsis* to drought stress conditions as simulated by exposure to mannitol.

### 2.6. Expression of Stress-Responsive Marker Genes Under Abiotic Stresses

**Figure 6:**
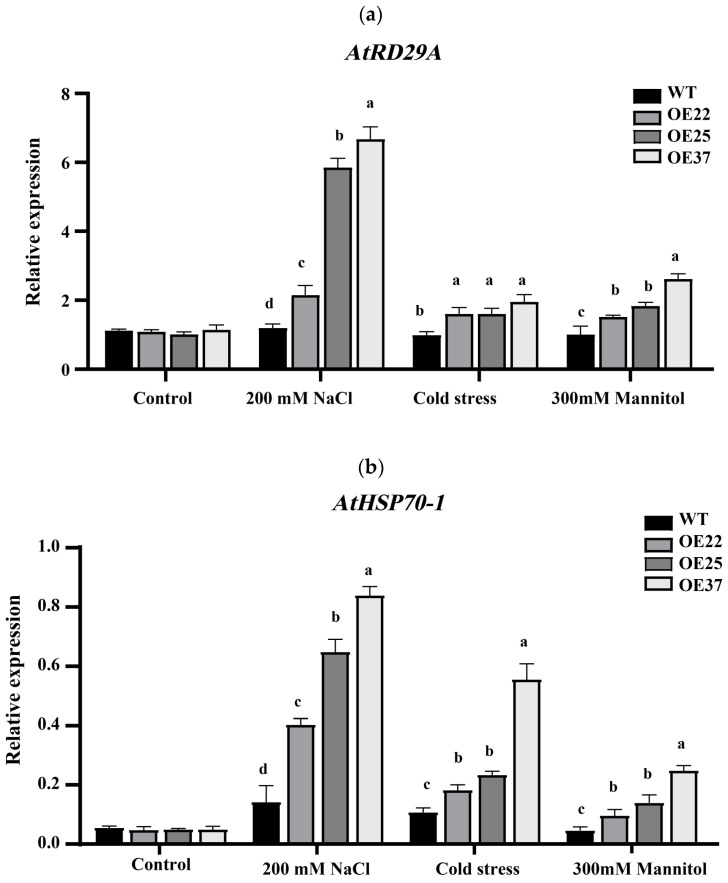
Expression of stress-responsive marker genes in WT and *TcDHN1*-OEs transgenic plants under abiotic stresses. qRT-PCR analysis of stress-responsive marker genes, including (**a**) *Arabidopsis AtRD29A* and (**b**) *Arabidopsis AtHSP70-1*. The expression level was normalized to that of *Arabidopsis AtACTIN*. The values are obtained from triplicate determinations and are represented as the means ± SEMs. The different letters above the bars specify significant differences (*p* < 0.05) as assessed with Tukey’s test.

To investigate whether *TcDHN1* overexpression modulates stress-responsive gene expression, the expression levels of related marker genes were analyzed. We selected the Response to Desiccation 29A gene (*AtRD29A*) and the Heat shock 70 kDa protein 1 (*AtHSP70-1*) gene to examine the transcript levels of these genes by qRT-PCR in WT and three *TcDHN1*-overexpressing lines (OE22, OE25, OE37) under both control conditions and various stress treatments. The results showed that, under control conditions, *AtRD29A* and *AtHSP70-1* transcript levels were comparable between WT and transgenic lines (Figure 6a,b). Upon stress treatment, both genes were significantly induced in all genotypes; however, the induction levels were markedly higher in *TcDHN1*-overexpressing lines than in WT across all three stress conditions. For instance, under salt stress, *AtRD29A* expression in OE lines ranged from approximately 1.8- to 5.5-fold higher than in WT, while *AtHSP70-1* expression ranged from 2.8- to 5.8-fold higher, with the highest induction observed in the OE37 line. Similar enhancement patterns were observed under drought and cold stresses. These results indicate that *TcDHN1* overexpression enhances the transcriptional activation of stress-responsive marker genes, suggesting that TcDHN1 potentiates plant stress responses, likely by modulating stress signaling pathways.

## 3. Discussion

Among all the known LEA proteins, DHNs constitute a distinct subset categorized under Group II ones [24,25]. They typically accumulate during seed maturation and in response to water deficits and desiccation induced by drought, low temperatures, and salinity [26], thereby playing a crucial protective role in plants. Their characteristic molecular features include evolutionarily conserved motifs, namely the lysine-rich K-segment, the N-terminal Y-segment, and frequently a phosphorylated S segment, as well as high hydrophilicity and an intrinsically disordered structure in the unbound state [27]. In this study, we successfully cloned the full-length CDS of *TcDHN1* from *T. chinensis*, which was 590 bp in size and showed high sequence homology with *DHN* genes from other plant species. Hydrophobicity analysis indicated that TcDHN1 was highly hydrophilic (Figure S1). Domain analysis revealed that the TcDHN1 protein contains two K segments and one S segment (Figure 1a). These structural properties are regarded as key factors in reducing water loss under stress conditions, which is equivalent to the role of the hydration buffer in maintaining water balance [28]. TcDHN1 was identified as an SK2 type DHN within the SKn subfamily, a group known to accumulate in response to low temperatures, salinity, dehydration and wound formation [29,30]. In line with this, the expression of *TcDHN1* was induced by various abiotic stresses, supporting its role in multiple stress tolerance, consistent with the findings on the *DHN* gene of *Sorghum bicolor* [31].

When plants are subjected to abiotic stresses such as drought, high salinity, and low temperatures, leading to cellular dehydration, proteins such as DHNs accumulate to high levels in various plant tissues and they are thought to play a role in abiotic stress tolerance [32]. The seeds of *T. chinensis* are recalcitrant and prone to inactivation under dehydration and low-temperature conditions, which represents a major bottleneck for its propagation and conservation [23,33]. Based on previous transcriptomic data of *T. chinensis* seeds under stress conditions, *TcDHN1* was found to be highly expressed [19]. Therefore, we focus here on TcDHN1 to explore its functional response to abiotic stress. In various species, the heterologous expression of DHNs has been shown to confer stress tolerance in plants [34,35,36]. In this study, overexpression of *TcDHN1* significantly enhanced salt, drought, and cold tolerance in *Arabidopsis*. Under salt stress, the transgenic lines exhibited significantly higher seed germination rates and longer root lengths compared to WT plants (Figure 3a,b). Similarly, under drought and cold stresses, the overexpression lines displayed improved survival rates and elevated activities of antioxidant enzymes, particularly SOD, CAT, and POD, which contributed to their enhanced performance under these adverse conditions (Figure 4 and Figure 5). These findings suggest that TcDHN1 may alleviate oxidative damage caused by diverse abiotic stresses by enhancing antioxidant enzyme activities, a function consistent with the previously reported roles of DHNs in stress protection [37,38]. However, its recalcitrant nature implies that the endogenous expression, regulation, and functional efficacy of *TcDHN1* may be insufficient during the critical phases of seed maturation and post-shedding.

Our findings are consistent with functional reports of *DHN* genes in multiple other species. For example, overexpression of the maize *ZmDHN15* gene enhanced cold tolerance in *Arabidopsis* [6], and overexpression of the soybean *GmDHN9* gene improved drought resistance in *Arabidopsis* [39]. In this study, *TcDHN1*-overexpressing lines exhibited enhanced tolerance to cold and drought stresses during seed germination, seedling emergence, and vegetative growth stages. It is noteworthy that the stress resistance conferred by DHN proteins from different origins may vary in strength. For instance, in this study, overexpression of *TcDHN1* improved the seed germination rates of *Arabidopsis* under 250 mM mannitol stress, whereas overexpression of the pepper *CaDHN5* gene, only conferred tolerance to 200 mM mannitol for seed germination [40]. Similarly, TcDHN1 overexpression enhanced root length tolerance in *Arabidopsis* to 200 mM mannitol, while overexpression of soybean *GmDHN9* enabled its tolerance to 300 mM mannitol [39]. These differences may stem from variations in amino acid sequences, domain compositions, post-translational modifications, and other factors among different DHN proteins, which collectively determine their biochemical properties and cellular protection efficiencies [41].

Abiotic stress often inflicts damage primarily through the excessive accumulation of ROS, which leads to oxidative damage [42]. ROS function as signaling molecules that regulate plant growth and responses to abiotic stress [43]. To maintain cellular homeostasis and reduce tissue damage, plants activate antioxidant systems, including SOD, CAT, and POD, to scavenge ROS [2]. The expression of DHNs has been shown to elevate the levels of antioxidant enzymes [44]. In our study, heterologous overexpression of *TcDHN1* in *Arabidopsis* significantly enhanced the activities of SOD, CAT, and POD under salt, drought, and cold stress, indicating that *TcDHN1* may help alleviate secondary oxidative damage [45]. This is consistent with previous reports that *DHN* transgenic plants exhibit enhanced stress tolerance by improving ROS scavenging ability [46]. The enhanced drought tolerance of *TcDHN1*-overexpressing lines was further supported by the upregulation of stress-responsive marker genes. *AtHSP70-1* functions as a molecular chaperone that responds to multiple abiotic stresses, including drought, salt, cold, and heat, by preventing protein misfolding, reducing aggregation of denatured proteins, and maintaining structural stability under adverse conditions [47,48], while *AtRD29A* is a well-established dehydration-responsive marker whose promoter contains dehydration-responsive elements (DRE) that specifically respond to drought, high salt, and cold stress [49]. These DRE cis-elements are recognized by DREB transcription factors, which regulate the expression of multiple downstream genes involved in stress tolerance [50]. Under stress conditions, transcript levels of *AtRD29A*, a classic marker of ABA-dependent stress signaling, and *AtHSP70-1*, a molecular chaperone involved in protein protection, were significantly higher in transgenic lines than in WT (Figure 6a,b). Consistent with their established roles in stress response pathways [47,51]. These results suggest that *TcDHN1* overexpression may enhance plant stress responses by modulating stress signaling pathways [32]. This study provides functional evidence that TcDHN1 confers tolerance to multiple abiotic stresses in *Arabidopsis*, likely through mitigating oxidative damage [52]. These insights establish TcDHN1 as a functional target for genetic improvement and provide a molecular rationale for future efforts aimed at enhancing its expression in *T. chinensis* seeds, once a stable transformation system becomes available. Further investigations should focus on dissecting the precise molecular pathways through which TcDHN1 operates, clarifying its role in stress responses and its potential for biotechnological applications.

## 4. Materials and Methods

### 4.1. Plant Materials

Mature recalcitrant seeds of the hemiparasitic plant, *T. chinensis*, were collected from the cultivation base of the Guangxi Botanical Garden of Medicinal Plants (23°09′ N, 108°21′ E) to serve as the plant materials for cloning of *TcDHN1* cDNA. For the overexpression analysis, the WT *Arabidopsis*, Columbia-0 (WT, Col-0), was used as the host for genetic transformation and functional analysis.

### 4.2. Nucleic Acid Extraction and cDNA Synthesis from Plant

Approximately 2.0 g of fresh transgenic *Arabidopsis* leaf tissue was rapidly ground into a fine powder in liquid nitrogen. Genomic DNA was extracted using the FastPure Plant DNA Isolation Mini Kit (Vazyme Biotech Co., Ltd., Nanjing, China). Total RNA was extracted using the FastPure Plant Total RNA Isolation Kit (Vazyme Biotech Co., Ltd., Nanjing, China). The purity and concentration of the nucleic acids were determined using a NanoDrop spectrophotometer (Thermo Fisher Scientific, Waltham, MA, USA). First-strand cDNA was synthesized from total RNA using the HiScript II 1st Strand cDNA Synthesis Kit (gDNA wiper) (Vazyme Biotech Co., Ltd., Nanjing, China). All DNA, RNA, and cDNA samples were stored at −80 °C for subsequent molecular analysis.

### 4.3. TcDHN1 Gene Sequences and Phylogenetic Analyses

The coding sequence of *TcDHN1* was amplified from *T. chinensis* cDNA using gene-specific primers (Table S1). The ORF finder in the NCBI database (http://www.ncbi.nlm.nih.gov/orffinder/) (accessed on 3 January 2026) was used to deduce the amino acid sequence it encodes. The physicochemical properties of the TcDHN1 protein were analyzed using the ExPASy ProtParam (https://web.expasy.org/protparam/) (accessed on 4 January 2026). DHN protein sequences from other species were downloaded from the NCBI website [53], and the sequence alignment of the DHN proteins was performed using DNAMAN software (version 6.0, Lynnon Biosoft, San Ramon, CA, USA). A phylogenetic tree of DHN proteins was constructed and finalized using MEGA (version 11.0) and iTOL (https://itol.embl.de/) (accessed on 9 March 2026) [54]. All the web pages and software information are listed in Table S1.

### 4.4. Vector Construction and Arabidopsis Transformation

The coding sequence (CDS) of *TcDHN1* was amplified from *T. chinensis* cDNA using gene-specific primers which incorporated *AbsI* and *KpnI* restriction sites (Table S1). The purified RT-PCR product was ligated into the linearized pCAMBIA1301 vector via homologous recombination, using the ClonExpress II One Step Cloning Kit (Vazyme Biotech Co., Ltd., Nanjing, China). The resulting recombinant plasmid, designated pCAMBIA1301-*TcDHN1*, was verified by restriction digestion and DNA sequencing, and then introduced into *Agrobacterium tumefaciens* strain GV3101 via the freeze–thaw method.

Transgenic *Arabidopsis* plants were generated the floral dip method [55]. *Agrobacterium* cultures carrying pCAMBIA1301-*TcDHN1* were resuspended in infiltration medium (5% sucrose and 0.02% Silwet L-77) to an OD_600_ of 0.6–0.8, and used to dip inflorescences of 3-week-old WT *Arabidopsis* (Col-0) plants. After infiltration, plants were kept in the darkness for 24 h and then returned to standard growth conditions (22 °C, 16 h light/8 h dark). T0 seeds were collected, surface-sterilized with 75% ethanol and 0.1% HgCl_2_, and sown on MS medium containing 30 mg·L^−1^ hygromycin. Resistant seedlings were transferred to soil and grown to maturity.

### 4.5. Identification and Selection of Transgenic Homozygous Lines

Collected T0 seeds were sown, and plants grown for approximately four weeks were designated the T1 generation. Genomic DNA was extracted from T1 plants and they were PCR amplified using *TcDHN1*-specific primers to identify positive transgenic individuals, with DNA from WT plants serving as a negative control. Segregation analysis of T1 progeny on selection medium was conducted to estimate transgene copy number. Putative positive plants were grown to maturity to produce the T2 generation.

To assess *TcDHN1* expression in T2 plants, total RNA was extracted from the leaves reverse-transcribed into cDNA, and analyzed by semi-quantitative RT-PCR (sqRT-PCR) using the *AtActin* as an internal reference gene. WT *Arabidopsis* cDNA and the recombinant plasmid DNA were used as negative and positive controls, respectively. For precise quantification, real-time quantitative PCR (qRT-PCR) was conducted using the QuantStudio 5 Real-Time PCR System (version 1.5.1, Thermo Fisher Scientific, Waltham, MA, USA) and the ChamQ Universal SYBR qPCR Master Mix (Vazyme Biotech Co., Ltd., Nanjing, China), by following the manufacturer’s instructions. Triplicate reactions were routinely performed per sample. The relative expression levels were calculated using the 2^−ΔΔCt^ method, with *AtActin* as the endogenous control. Three transgenic lines exhibiting the highest accumulation of *TcDHN1* transcripts were selected via qRT-PCR. These high-expressing lines were self and propagated samples to obtain homozygous overexpression lines for subsequent functional assays. All the primer sequences used are listed in Table S1.

### 4.6. Abiotic Stress Treatments on WT and Transgenic Arabidopsis

The abiotic stress tolerance of WT and three homozygous *TcDHN1*-OEs lines was evaluated under salt, cold, and drought conditions. For each assay, the specific stress agents and their concentrations were selected based on preliminary experiments and previously established protocols for Arabidopsis stress tolerance studies [56,57].

Seeds were stratified at 4 °C for 2 days and surface-sterilized with 0.1% HgCl_2_, and then plated onto MS solid media containing different stress agents: salt stress (100 and 150 mM NaCl), drought stress (simulated with 150 and 250 mM mannitol), and using normal MS media as a control. All plates were incubated under standard growth conditions (22 °C, 16 h light/8 h dark). For cold stress, seeds were plated onto MS media and placed at 4 °C, with those grown at 22 °C serving as the unstressed control under the same photoperiod regimen (16 h light/8 h dark). Germination rates were recorded periodically. Each treatment consisted of 30 seeds and was performed in triplicate.

One-week-old seedlings grown on MS media, with a primary root length of approximately 1 mm and uniform growth, were transferred to 1/2 MS solid media containing the stress agents: salt stress (50 and 100 mM NaCl), drought stress (150 and 200 mM mannitol), with normal 1/2 MS media as a control. Plates were maintained under standard conditions for 10 days before their root lengths. For cold stress, seedlings on normal 1/2 MS media were transferred to a 4 °C environment, with seedlings kept at 22 °C serving as the unstressed control under the same photoperiod for 14 days before measuring their root lengths. Each treatment consisted of 5 seedlings and was performed in triplicate.

For soil-grown plants, three-week-old, uniformly developed WT Arabidopsis and transgenic seedlings were subjected to abiotic stress treatments. Salt and drought stresses were performed by irrigating with 200 and 300 mM NaCl as well as 300 and 400 mM mannitol solutions, respectively. For cold stress, whole plants were transferred to a 4–growth chamber (16 h light/8 h dark) with untreated plants maintained under standard conditions to served as the control. Growth phenotypes were documented after 1–2 weeks of treatment and each was performed in triplicate.

### 4.7. Measurement of Antioxidant Enzyme Activities

To assess the oxidative stress response, seedlings of WT and three homozygous *TcDHN1*-OEs lines, were harvested after 1–2 weeks under salt, cold, and drought stress conditions. The fresh weights of the collected seedlings were recorded immediately. For enzymatic assays, the seedlings were homogenized in ice-cold phosphate buffer (pH 7.4) and centrifuged at 12,000× *g* for 15 min at 4 °C. The supernatants were collected, and the total protein concentration was determined using the Bradford assay [58]. Subsequently, the activities of the antioxidant enzymes superoxide dismutase (SOD), catalase (CAT), and peroxidase (POD), were determined using corresponding assay kits (Servicebio, Wuhan, China) according to the manufacturer’s instructions.

### 4.8. Expression Analyses of Relevant Marker Genes

To analyze the expression of stress-responsive marker genes in WT and *TcDHN1*-overexpressing lines under abiotic stresses, leaves of two-week-old Arabidopsis seedlings were collected after 24 h of salt (200 mM NaCl), drought (300 mM mannitol), or cold (4 °C) treatment. Total RNA was extracted from leaves using the FastPure Plant Total RNA Isolation Kit (Vazyme Biotech Co., Ltd., Nanjing, China) in accordance with the manufacturer’s instructions, and qRT-PCR analysis was performed to examine the expression of *AtRD29A*, and *AtHSP70-1*. The specific primers are listed in Table S1. *AtActin* (Table S1) was used as the internal reference gene to analyze the transcript levels of the marker genes. Relative expression levels were calculated using the 2^−ΔΔCt^ method. Three independent biological experiments were performed, each with three technical replicates.

### 4.9. Statistical Analysis

All the data are presented as means of triplicate measurements. Statistical significance was determined using Tukey’s test for multiple comparisons. In all analyses, *p* values of <0.05 were considered statistically significant. Analyses were conducted using Microsoft Excel and SPSS (version 24, Chicago, IL, USA).

## 5. Conclusions

This study identified *TcDHN1* as an SK2-type dehydrin from the recalcitrant seeds of *T. chinensis* and demonstrated its function as a positive regulator of abiotic stress tolerance, likely through mitigating oxidative damage. These findings expand the functional repertoire of dehydrins in medicinal plants and highlight *TcDHN1* as a potential genetic target for improving stress resistance and seed desiccation tolerance. A limitation of this work is the reliance on heterologous overexpression; thus, future efforts should focus on in planta functional validation in *T. chinensis* using virus-induced gene silencing (VIGS) or CRISPR/Cas9-mediated editing. Furthermore, elucidating the expression dynamics of *TcDHN1* throughout seed development and in response to dehydration will be critical for understanding its contribution to the recalcitrant seed phenotype and for designing targeted interventions to improve future seed storability.

## Data Availability

The original contributions presented in this study are included in the article/Supplementary Material. Further inquiries can be directed to the corresponding authors.

## Supplementary Materials

The following supporting information can be downloaded at: https://www.mdpi.com/article/10.3390/plants15060884/s1, Figure S1: The full-length CDS of *TcDHN1* was cloned from *T. chinensis*. (A) Extraction of the total RNA from *T. chinensis*; (B) RT-PCR product of *TcDHN1*. M: DNA markers; 1, 2 and 3: RT-PCR products of *TcDHN1*; Figure S2: Nucleotide and amino acid sequences of *TcDHN1*; Figure S3: Hydrophobicity analysis (the positive peak value represents hydrophobicity, and the negative peak value represents hydrophilicity); Figure S4: Construction of the *TcDHN1* Overexpression Vector. (A) Schematic of the recombinant vector pCAMBIA1301-*TcDHN1* construction; (B) PCR amplification of the TcDHN1 gene; (C) Restriction digestion analysis of pCAMBIA1301-*TcDHN1*; M: DNA Markers (DL2000); Lanes 1–3: Parallel samples, all showing the *TcDHN1* gene amplification product; Lane 4: Circular plasmid of pCAMBIA1301-*TcDHN1*; Lane 5: Restriction digestion products of the pCAMBIA1301-*TcDHN1* plasmid from a positive clone; Table S1: The primers used in this study.
