# Supplementary material for: The Enhancement of Abiotic Stress Tolerance in Arabidopsis via Heterologous Overexpression of TcDHN1, a Dehydrin Identified in the Recalcitrant Seeds of Taxillus chinensis
Source: Plants (Basel). 2026 Mar 12;15(6):884. doi: 10.3390/plants15060884 (PMC13030604; doi:10.3390/plants15060884)
Supplement: Supplementary file 1 [file plants-15-00884-s001.zip › Supplementary_Figure.pdf]

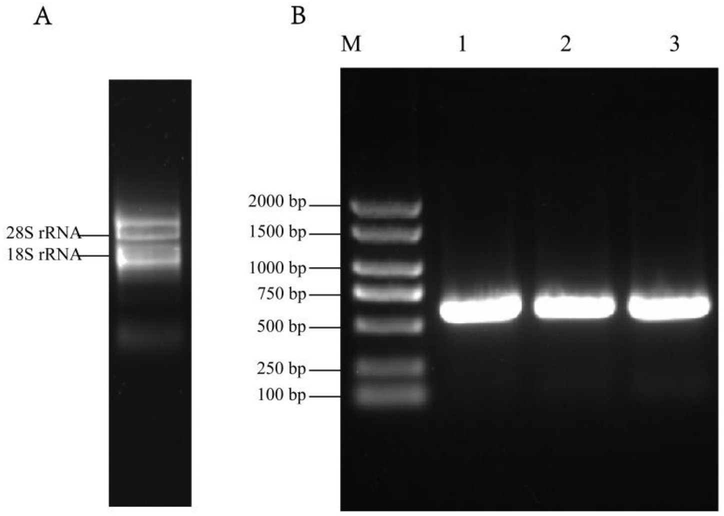

Figure S1. The full-length CDS of *TcDHN1* was cloned from *T. chinensis.* (A) Extraction of the total RNA from *T. chinensis*; (B) RT-PCR product of *TcDHN1.* M: DNA markers; 1, 2 and 3 : RT-PCR product of *TcDHN1*.

```
                  10         20         30         40         50         60
1        ATGGCCGAAGAAAATCCTCATCCCGCCGTCGCCGCCGGAGAGGCCGCCGTGGAGACCAAA
1         M  A  E  E  N  P  H  P  A  V  A  A  G  E  A  A  V  E  T  K

                  70         80         90        100        110        120
61       GATCGGGGGTTGTTTGAACGATTCTTGGGCAAGGACGAGAAGGAGAAGCACGAGGAGGAG
21        D  R  G  L  F  E  R  F  L  G  K  D  E  K  E  K  H  E  E  E

                 130        140        150        160        170        180
121      GCGATTGCCTCCGATTTGGAGAAGGTACACGTCTCCGAGCCCGACAAGCCGACAGAGAAA
41        A  I  A  S  D  L  E  K  V  H  V  S  E  P  D  K  P  T  E  K

                 190        200        210        220        230        240
181      GAAGAAAAAGAAAAACACTCTACTCTGTTGGAGAAGCTTCACCGATCTAATAGCAGCTCA
61        E  E  K  E  K  H  S  T  L  L  E  K  L  H  R  S  N  S  S  S

                 250        260        270        280        290        300
241      AGCTCATCTAGTGATGAGGAGCAGGGAGATGGAGGAGAGAAGAAGAAGAAGAAAAAGAAG
81        S  S  S  S  D  E  E  Q  G  D  G  G  E  K  K  K  K  K  K  K

                 310        320        330        340        350        360
301      GGATTGAAGGAGAAAGTTAGTGGAGAGGAGGAAGTGAAGGTGGAGGATACCACCGTCCAG
101       G  L  K  E  K  V  S  G  E  E  E  V  K  V  E  D  T  T  V  Q

                 370        380        390        400        410        420
361      ATCGAAAAGTACGAGGAATCGGAGGCGCCGGAGGGAGCGGAGAAAAAGGGTCTCATAGAA
121       I  E  K  Y  E  E  S  E  A  P  E  G  A  E  K  K  G  L  I  E

                 430        440        450        460        470        480
421      AAGATTAAGGAAAAACTTCCAGGACACAACAAGAAGGCTGAGGAGCATCCTCCGCCGGCT
141       K  I  K  E  K  L  P  G  H  N  K  K  A  E  E  H  P  P  P  A

                 490        500        510        520        530        540
481      GCCCTGGAGTCTGCCGCGGTTGAGGCTGGTCATGAGGGCGAAGGAAAAGAGAAGAAGGGC
161       A  L  E  S  A  A  V  E  A  G  H  E  G  E  G  K  E  K  K  G

                 550        560        570        580        590
541      GTTTTCGACAAGATAAAGGAAAAAATCCCAGGGTACCCAAGAGTGAATGA
181       V  F  D  K  I  K  E  K  I  P  G  Y  P  R  V  N
```

Figure S2. Nucleotide and amino acid sequences of *TcDHN1*

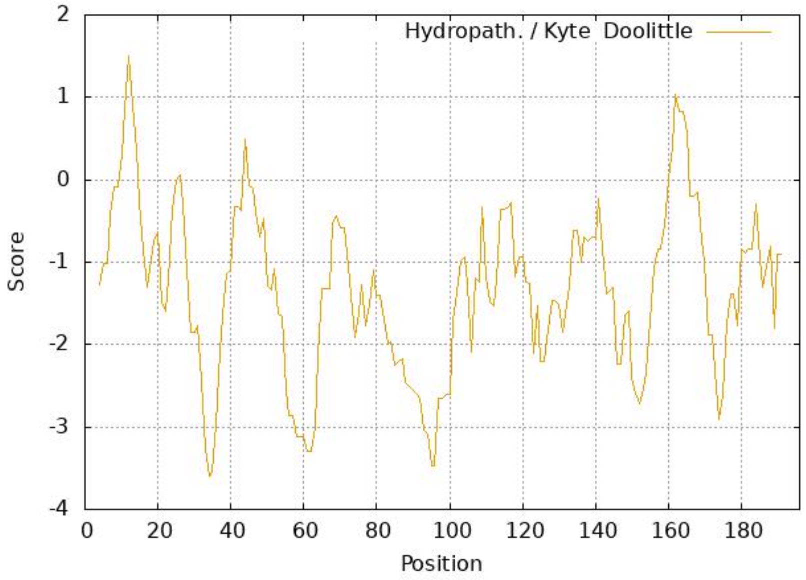

Figure S3. Hydrophobicity analysis (the positive peak value represents hydrophobicity and the negative peak value represents hydrophilicity).

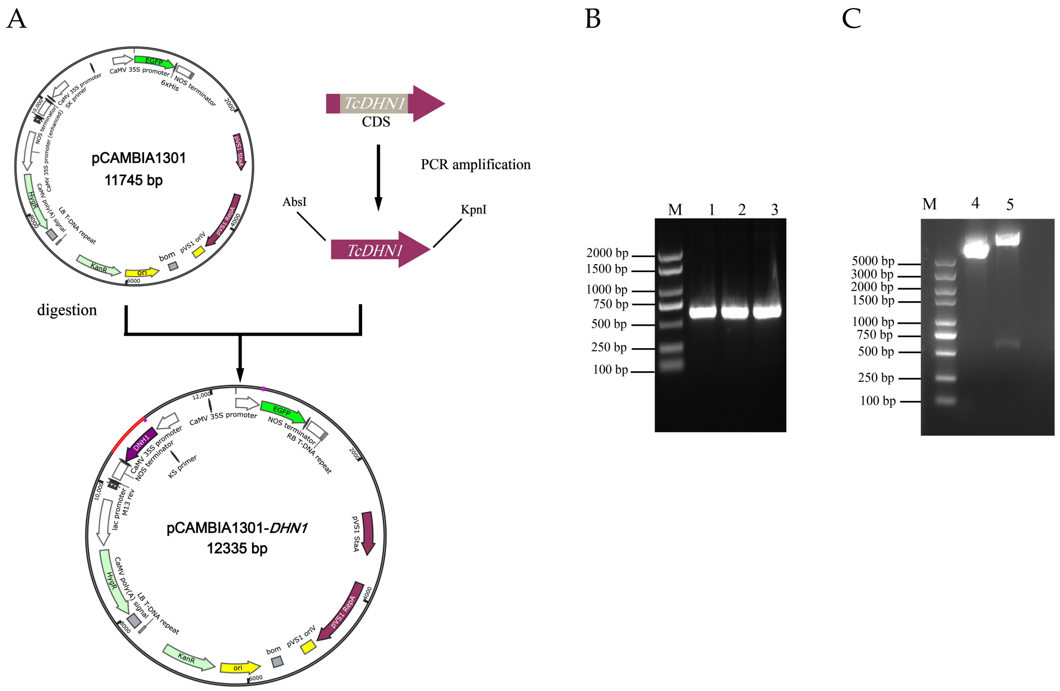

Figure S4. Construction of the *TcDHN1* Overexpression Vector. (A) Schematic of the recombinant vector pCAMBIA1301-*TcDHN1* construction; (B) PCR amplification of the *TcDHN1* gene; (C) Restriction digestion analysis of pCAMBIA1301-TcDHN1, M: DNA Markers (DL2000), Lanes 1-3: Parallel samples, all showing the *TcDHN1* gene amplification product, Lane 4: Circular plasmid of pCAMBIA1301-*TcDHN1*, Lane 5: Restriction digestion products of the pCAMBIA1301-*TcDHN1* plasmid from a positive clone.
